# Enhancing social communication behaviors in children with autism: the impact of dog training intervention on verbal and non-verbal behaviors

**DOI:** 10.3389/fpsyg.2024.1496915

**Published:** 2024-12-23

**Authors:** Yaara Polak-Passy, Esther Ben-Itzchak, Ditza A. Zachor

**Affiliations:** ^1^Department of Communication Disorders, Bruckner Autism Research Center, Ariel University, Ariel, Israel; ^2^The Autism Center, Department of Pediatrics, Assaf Harofeh Medical Center, Sackler Faculty of Medicine, Tel Aviv University, Zerifin, Israel

**Keywords:** autism spectrum disorders, dog training intervention, animal assisted intervention (AAI), non-verbal behaviors, verbal behaviors, maladaptive behaviors

## Abstract

**Introduction:**

This study investigated the impact of dog training intervention (DTI) on verbal, non-verbal, and maladaptive behaviors in autistic preschoolers. Previous research has demonstrated the benefits of animal-assisted interventions, but this study specifically focused on changes during the DTI.

**Methods:**

We analyzed video recordings of 37 autistic children (mean age 4:7 years, SD = 1:1) from special education preschools, comparing their behaviors during the initial and final intervention sessions. The intervention, consisting of 17 structured stages, progressively introduced the children to dog interactions, ultimately allowing them to act as dog trainers. Behavioral analysis was divided into two main areas: responses to the therapist’s instructions and self-initiated behaviors observed in interactions with the therapist and the dog.

**Results:**

Post-intervention results indicated a notable increase in non-verbal communication, with more sustained self-initiated eye contact, gestures, and facial expressions and increased verbal commands directed toward the dog. Improvements were also seen in therapist-prompted gestures and joint attention, and question-answer interactions with the therapist. However, a decrease was observed in self-initiated eye contact, duration of eye contact, and verbal sharing with the therapist. Maladaptive behaviors, such as inappropriate physical contact and repetitive movements, decreased. The study found a moderate negative correlation between autism severity and responsiveness to therapist instructions and a moderate positive correlation between IQ and improvements in therapist responsiveness.

**Discussion:**

These findings support the growing evidence for the efficacy of dog-assisted interventions and emphasize the importance of tailoring interventions to individual child characteristics.

## 1 Introduction

Autism spectrum disorder (ASD) is marked by challenges in social communication and repetitive behaviors that significantly affect individuals and their families (American Psychiatric Association, 2013). The research underscores the necessity of interventions that target developmental and functional skills in autism, highlighting their potential to enhance social and adaptive abilities (Ben-Itzchak et al., 2014; Ben-Itzchak and Zachor, 2011; Dawson et al., 2010).

Social—communication behaviors were previously investigated in various studies and highlighted distinct differences in social communication styles between autistic and non-autistic children, particularly regarding their use of verbal and non-verbal communicative gestures. While children with ASD often use gestures such as pointing or conventional signs in early interactions, they generally show a reduced tendency to use these gestures to share attention, although they may still use them to make requests (Hurwitz and Watson, 2016; LeBarton and Iverson, 2016). This reduced use of joint attention gestures, such as pointing to share an experience, has been linked to challenges in developing social engagement skills and is a predictor of concurrent and future language abilities (Valle et al., 2021). Specifically, Valle et al. (2021) examined communicative gestures in minimally verbal children and adolescents with ASD, finding that these gestures often serve as a crucial complement to spoken language abilities, especially in social contexts. In their study, while children with ASD used gestures to supplement limited verbal communication, the function and frequency of these gestures were less socially oriented compared to their non-autistic peers. These findings suggest that understanding the nuanced use of gestures in ASD is essential, as these behaviors are key predictors of later language and social development. Building on this understanding, the current study focuses on assessing specific changes in non-verbal and verbal behaviors in the context of dog-assisted intervention, which is part of animal-assisted Intervention (AAI).

In recent years, animal-assisted intervention (AAI) has gained traction as a supplementary treatment across various medical and developmental challenges (Silva et al., 2011). Studies focused on autism suggest substantial benefits from integrating AAI into personalized treatment plans. These include enhanced social skills and verbal and non-verbal communication, reductions in negative behaviors such as aggression and obsession (Redefer and Goodman, 1989) and increases in language use and social interaction noted in school-based occupational therapy settings (Sams et al., 2006). Furthermore, AAI has been associated with greater social engagement, increased calmness (Martin and Farnum, 2002; Silva et al., 2018), and the reduction of sensory avoidance behaviors through the multisensory stimulation provided by a dog’s presence (Redefer and Goodman, 1989).

Recent systematic reviews by Rehn et al. (2023), Nieforth et al. (2023), O’Haire (2013, 2017) highlight the potential benefits of AAI for children and adolescents with ASD while also underscoring variability in outcomes. Across these reviews, positive trends are observed in social, physical, and functional domains, with social engagement and communication skills showing the most consistent improvements, such as increased frequency of interactions and reduced social withdrawal. Emotional and behavioral outcomes, like anxiety reduction and mood regulation, also improved in some studies, though these effects were more variable.

Together, these reviews highlight the need for consistent frameworks in AAI research, as methodological heterogeneity—stemming from differences in intervention types, animal species, and outcome measures—creates challenges in interpreting results. Despite these limitations, the growing body of evidence supports AAI as a potentially effective approach to enhancing social and functional skills in children and adolescents with ASD, with promising adaptability across cultural contexts and various animal species. These reviews examined interventions involving various animals, such as dolphins, horses, and dogs, further complicating comparisons across studies.

Interaction with dogs was previously reported as beneficial for individuals with ASD. Research suggests several benefits of dog ownership for individuals with autism, particularly in social and emotional domains. Barcelos et al. (2021) indicate that dogs provide a consistent source of non-judgmental companionship, helping to alleviate loneliness and enhance social engagement. Additionally, dogs may promote mental health stability by reducing anxiety and depressive symptoms, creating a positive impact on overall well-being and potentially acting as protective factors in suicide prevention (Wright et al., 2021).

Focusing on interventions that used dogs, interactions with them were associated with significant improvements in social engagement and communication and reduced self-directed behaviors. Redefer and Goodman (1989) reported a substantial increase in prosocial behaviors, such as social interaction with the therapist and dog, and a concurrent decrease in self-absorption and stereotypical behaviors in children aged 5–10. Martin and Farnum (2002) further confirmed these benefits, observing enhanced focus, social awareness, and playful mood in children aged 3–13 when interacting with a live dog compared to a ball or stuffed animal. However, some challenges, such as increased hand-flapping and reduced eye contact, were noted in the “dog” condition. Fung and Leung (2014) similarly found that children engaged in more verbal social behaviors when interacting with a dog compared to a doll. Germone et al. (2019) also demonstrated that interactions with dogs significantly increased social communication among hospitalized youth with ASD compared to interactions with a novel toy. Additionally, Ávila-Álvarez et al. (2020) observed notable gains in non-verbal communication and social skills, including eye contact and physical interaction, during a dog-assisted intervention for children aged 2.5–6. Overall, these findings suggest that dog-assisted interventions can effectively enhance social engagement and communication in children with ASD, with varying levels of improvement in different contexts and age groups. Barcelos et al. (2021) indicate that dogs provide a consistent source of non-judgmental companionship, helping to alleviate loneliness and enhance social engagement. Additionally, dogs may promote mental health stability by reducing anxiety and depressive symptoms, creating a positive impact on overall well-being and potentially acting as protective factors in suicide prevention (Wright et al., 2021).

Building on this research, our initial study (Ben-Itzchak and Zachor, 2021) explored the impact of a “Dog Training Intervention” (DTI) using a structured protocol within special education settings for autism. This controlled crossover study evaluated changes in adaptive skills, autism severity, and anxiety symptoms, revealing significant enhancements in adaptive social and communication skills among participants who underwent DTI. These improvements persisted post-DTI. Better outcomes were linked to initial group assignment to DTI, higher pre-intervention adaptive skills, superior baseline cognitive abilities, and less prominent autistic characteristics. However, only a few previous studies have assessed changes in specific social communication and stereotypical behaviors following interventions with dogs in populations diagnosed with autism.

The current study expands on our previous research by examining changes in specific social-communication behaviors and aberrant behaviors following the DTI program. Our aims include assessing changes in communication behaviors, evaluating maladaptive behaviors, and examining the correlation between children’s characteristics and progress in social-communication behaviors during intervention sessions from the beginning to the end of the intervention period.

The study’s aims include: 1. Assessing changes in non-verbal and verbal communication behaviors addressed toward the dog, either in response to the therapist’s instructions or self-initiated interactions. Non-verbal behaviors include the number of eye contact, facial expressions, and gestures events, as well as the mean eye contact duration. Verbal behaviors mainly consist of commands to the dog. 2. Assessing changes in non-verbal and verbal communication behaviors addressed to the therapist, either in response to the therapist’s instructions or self-initiated interactions with the therapist. Non-verbal behaviors include the number of eye contact, facial expressions, and gestures events, and the mean duration of eye contact. Verbal behaviors encompass statements, questions/responses, requests, refusal, and verbal sharing. 3. To Assess changes in maladaptive behaviors, such as inappropriate physical contact, facial expressions and sensory behaviors, repetitive motor movements, and use of objects. 4. To examine the correlation between children’s characteristics (i.e., age, cognitive ability, the intensity of autism characteristics, and level of anxiety) and progress in social—communication behaviors after DTI in two conditions, response to the therapist’s instructions and self-initiated behaviors. Chat GPT-4 software was used solely for linguistic editing purposes.

## 2 Methods

### 2.1 Dog training intervention

The intervention was facilitated by “Dogs for People,” a non-profit organization, with certified dog therapists trained at Achva Academic College. The program spanned 4 months, comprising bi-weekly sessions with a therapist-to-child ratio of 1:1 or 2:2. Session durations varied, starting and ending with 45-min sessions and 20-min sessions in the middle. Six mixed-breed dogs, aged between one and 2 years, selected for their temperament and obedience, were involved in the program. Initially, all dogs underwent suitability tests to ensure they were not sensitive to pulling and exhibited positive behavior around people, other dogs, and cats. After passing these tests, the dogs participated in a basic obedience training series, during which they learned to recognize both verbal and physical commands. In the final stage of training, the dogs were prepared to become agile performers. By the end of this process, they were trained in the canine sport of “dancing with dogs” and could perform various tricks. After successfully completing all these training stages, the dogs were only assigned to work with children and adults. The training involved 17 specific stages aimed at gradually acclimating children to interactions with dogs, ultimately enabling them to engage as dog trainers. The following targets were included in the intervention; some were divided into several stages: 1. *Adjustment to the dogs:* The dogs walked among the children, around the school, and in the yard without requiring the children to interact. This stage lasted approximately 2 weeks. 2. *First physical contact with dogs:* The children began by touching or petting the dogs, starting from the tail and moving toward the head. 3. *Feeding the dogs with a spoon:* The children fed the dogs using a spoon without touching them. 4. *Walking the dogs with a leash:* Initially, the trainer held the leash with the child, gradually letting the child walk the dog alone. Over time, the leash length was shortened, and the duration of the walks was extended. 5. *Learning to communicate with the dogs*: The children learned to give commands using gestures, words, and proper intonation. They observed how integrating these communication components improved the dogs’ responsiveness. The children also learned to give positive reinforcement, such as treats or verbal praise, for obeying commands. 6. *Two children walking one dog with two leashes*: This activity required the children to coordinate and communicate with each other. 7. *Advanced command giving:* The children learned to give commands that combined coordinated words, gestures, and movements (e.g., commanding “jump” while running). The transition was made to more energetic dogs during this stage, and equipment like springboards and cones were introduced. 8. *Complex commands:* The children learned to give commands of two or more parts. 9. *Independent initiation:* The children began independently interacting with the dogs in group work. All sessions were video-documented.

### 2.2 Measures

*Social Responsiveness Scales-2 (SRS-2):* This 65-item scale, developed by Constantino and Gruber (2012), relies on observer ratings to assess traits associated with autism. The responses are recorded on a 4-point Likert Scale (0–3 points). The SRS-2 provides a total score and two primary indices reflecting the main symptom domains of ASD: Social Communication and Interaction (SCI) and Restricted Interests and Repetitive Behavior (RIRB). Scores are standardized (*M* = 50, SD = 10), with higher scores indicating more severe autistic traits. Scores ranging from 60 T to 65 T suggest mild severity, 66–75 T indicate moderate severity, and 76 T or higher reflect severe symptoms. Scores of 65 T and above are considered clinically significant.

*Wechsler Preschool and Primary Scale of Intelligence—Third Edition (WPPSI-III HEB)* (Wechsler, 1989). An intelligence assessment for children aged 2:6 to 7:3 years, administered by trained psychologists. It comprises four subscales: Verbal IQ, Performance IQ, Processing Speed, and Full-Scale IQ, with all indices reported as standard scores (*M* = 100, SD = 15).

*Spence Children’s Anxiety Scale (SCAS)* (Spence et al., 2001): This scale measures self-reported anxiety symptoms in children, consisting of 44 items rated on a 4-point scale from ‘never’ (0) to ‘always’ (3). It includes six subscales corresponding to different types of anxiety as described in DSM-IV: panic/agoraphobia, separation anxiety, social phobia, generalized anxiety, obsessive-compulsive anxiety, and fear of physical injury. The SCAS has demonstrated strong psychometric properties with normative data indicating mean scores of 18.81 (SD = 10.90) for 4-year-olds and 18.27 (SD = 12.23) for 5-year-olds (Spence et al., 2003). Cronbach’s alpha for the SCAS ranges from 0.86 to 0.94.

*Video analysis:* Therapy sessions were recorded, each participant contributing several hours of footage. Following previous studies’ methodology (Martin and Farnum’s, 2002; O’Haire et al., 2013; Germone et al., 2019) the recordings were edited to produce two 3-min videos per participant, representing the start and end of the intervention sessions. Video editing focused on capturing the most illustrative angles to observe the child’s behaviors. Approximately 60% of the videos were edited for 3 preferred by the first author (Y.P.), with the sole criterion being the quality of the footage and the ability to identify the desired behaviors. Of note, Y.P. was not part of the original research. When editing the videos, she was not blinded to the study conditions, and the editing was performed to identify relevant material for the research. The remaining videos (40%) were edited by a research assistant who was blinded to the study’s objectives and conditions of the videos she edited.

*Assessment of verbal, non-verbal, and maladaptive behaviors:* The participants’ verbal and non-verbal behaviors toward the dog and the therapist were assessed during the intervention. Behaviors were classified and analyzed separately based on whether they were initiated by the participant or resulted from the therapist’s instructions.

Non-verbal behaviors included the number of eye contact events, gestures, and facial expressions directed and undirected at others. The number of joint attention events toward the therapist was also counted. Verbal behaviors directed at the therapist included the number of statements, questions/responses, requests, refusals, and verbal sharing. Additionally, the number of verbal commands the participants gave to the dog was recorded.

Maladaptive behaviors included inappropriate physical contact, facial expressions and sensory behaviors, repetitive motor movements, and use of objects.

*Video coding and reliability*: Video coding was conducted to evaluate verbal and non-verbal behaviors toward the therapist and the dog separately pre- and post-intervention. This was performed by the author (Y.P), a speech and language pathologist (SLP) experienced in ASD. To ensure coding reliability, 37% of the videos were independently coded by another SLP, who was blind to the therapy phase. An inter-coder reliability of 91% was achieved. The Observer XT software, designed for video analysis and coding, was utilized in this process.

### 2.3 Participants

In the original study (Ben-Itzchak and Zachor), all parents of children attending 10 special preschools for children with ASD were invited to participate in the original study. Out of 74 children, only one was excluded due to parental refusal. Therefore, all 73 remaining children participated in the study, with no other exclusion criteria applied. The study involved 37 out of the original 73 participants who were selected based on the quality of the videos (see the “video analysis” section). To ensure that the group participating in the current study was comparable to those excluded from the initial study due to limitations in video recordings, we conducted a comparison of age, gender, autism severity (using SRS II scores), IQ scores (via ANOVA), and adaptive skills (assessed through VABS subdomain scores using MANOVA) between the two groups. No differences were found between the participants in the current study and the group of children who did not participate in this study on any of these measures.

The current study included 33 boys and four girls, aged between 2:11 and 6:11 years (*M*_age_ = 4:7 years, SD 1:1 year). All participants resided in an area with a medium-high socioeconomic status. Each participant had been clinically diagnosed with ASD through comprehensive medical and psychological assessments in line with DSM-5 criteria. Participants were recruited from 10 specialized preschools for autistic children. Eligibility was determined based on recognition by the National Insurance Institute, which granted access to these preschools. The study obtained ethical approval from the Governmental Department of Education. Parents provided written informed consent for their children’s participation and data use, following the requirements of the ethical committee.

### 2.4 Statistical analysis

Data were collected at two time points: T1 (beginning of intervention) and T2 (end of intervention). Analyses included 2 × 2 MANOVAs with repeated measures for time (T1/T2) and communication modes (response to therapist’s instructions/participant-initiated), examining verbal and non-verbal behaviors during sessions. Several 2 × 2 ANOVAs for each dependent variable followed significant MANOVA results. Simple main effects for Time were analyzed when the Time × Communication mode interaction was significant. Additionally, 2 × 2 ANOVAs were conducted for the duration of eye contact with the dog and the therapist as dependent variables. Pearson correlation analyses were performed to examine the relationship between the verbal and non-verbal behaviors change from T1 to T2 and the baseline scores of SRS-II, WPPSI-III, and SCAS.

## 3 Results

### 3.1 The size of the study population

Since participant selection was based on video quality, determining the number of participants in advance was not feasible. Consequently, a *post hoc* G*Power analysis was conducted to verify that the statistical power in the analyses was adequate. A *post hoc* G*Power analysis with an effect size of 0.3, an alpha level of 0.05, and a correlation between variables of 0.4 (mean of the repeated measures correlations) indicated a statistical power of 0.90 which ensures that the findings are robust and that there is a sufficiently high probability of detecting true effects, if present.

### 3.2 Behaviors toward the dog

#### 3.2.1 Non-verbal behaviors

First, we compared non-verbal behaviors at the commencement and conclusion of DTI across two distinct behavioral contexts: in response to the therapist’s instructions and during interactions initiated by the participant with the dog. These behaviors encompassed the frequency of eye contact events, occurrences of gestures, and directed and undirected facial expressions toward the dog. A 2 × 2 MANOVA [2 times (T1/T2) × 2 communication modes (response to therapists’ instructions; participant-initiated)] with repeated measures for time revealed several significant effects. Specifically, there was a significant time effect (*F*(4,33) = 9.66, *p* < 0.001, *η*^2^ = 0.54), behavior mode effect (*F*(6,31) = 18.25, *p* < 0.001, *η*^2^ = 0.69), and a significant time x behavior mode interaction (*F*(6,31) = 5.58, *p* = 0.02, *η*^2^ = 0.40).

**Figure 1:**
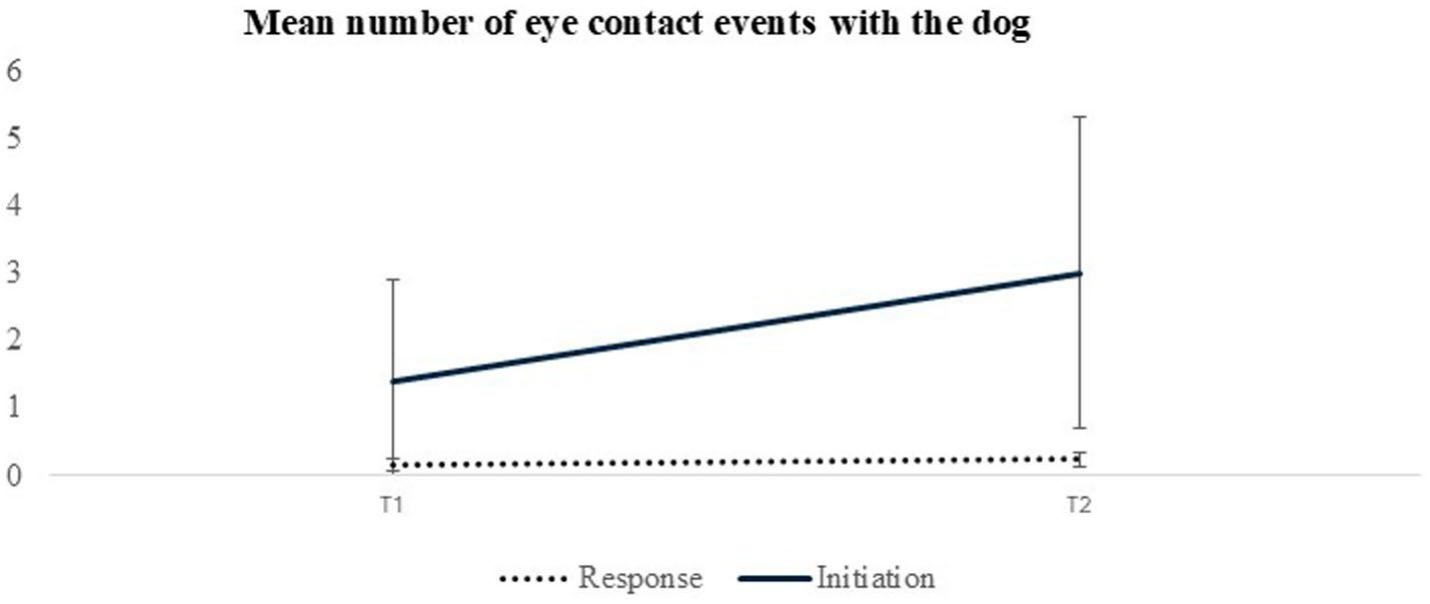
Mean number of eye contact events with the dog in response to therapists’ instructions and for participant-initiated eye contact events at the start and the end of DTI.

Subsequent 2 × 2 ANOVAs, with repeated measures for time conducted separately for each behavior, indicated that the interaction was significant only for the number of eye contact events (Figure 1).

Further analyses to explore this interaction revealed a significant time effect (*F*(1,36) = 21.47, *p* < 0.001, *η*^2^ = 0.35) for participant-initiated behaviors but not for behaviors directed to the dog in response to the therapists’ instructions. By the program’s conclusion, there was a notable increase only in the number of eye contact events initiated by the participants toward the dog. Additionally, subsequent one-way ANOVAs for each non-verbal behavior revealed a significant time effect for the number of eye contacts, gestures, and undirected appropriated facial expression events, indicating increased frequencies of these behaviors observed after completing the DTI (Table 1).

**Table 1:** Mean counts of non-verbal and verbal communication events directed toward the dog at the start (T1) and end (T2) of DTI.

	T1	T2	*F*(1,36)	*p*	*χ* ^2^
	*M* (SD)	*M* (SD)			
Non-verbal behaviors					
Eye contact	0.78 (0.13)	1.64 (0.23)	**20.05**	**<0.001**	0.37
Gestures	0.53 (0.11)	2.12 (0.31)	**24.35**	**<0.001**	0.42
Directed appropriate facial expressions	0.22 (0.07)	0.23 (0.06)	0.02	0.88	0.001
Indirect appropriate facial expression	0.28 (0.06)	0.14 (0.04)	**3.32**	**0.007**	0.08
Verbal communicative behavior					
Command	0.37 (0.13)	2.75 (3.51)	**38.9**	**<0.001**	0.52

Values in bold are statistically significant.

**Figure 2:**
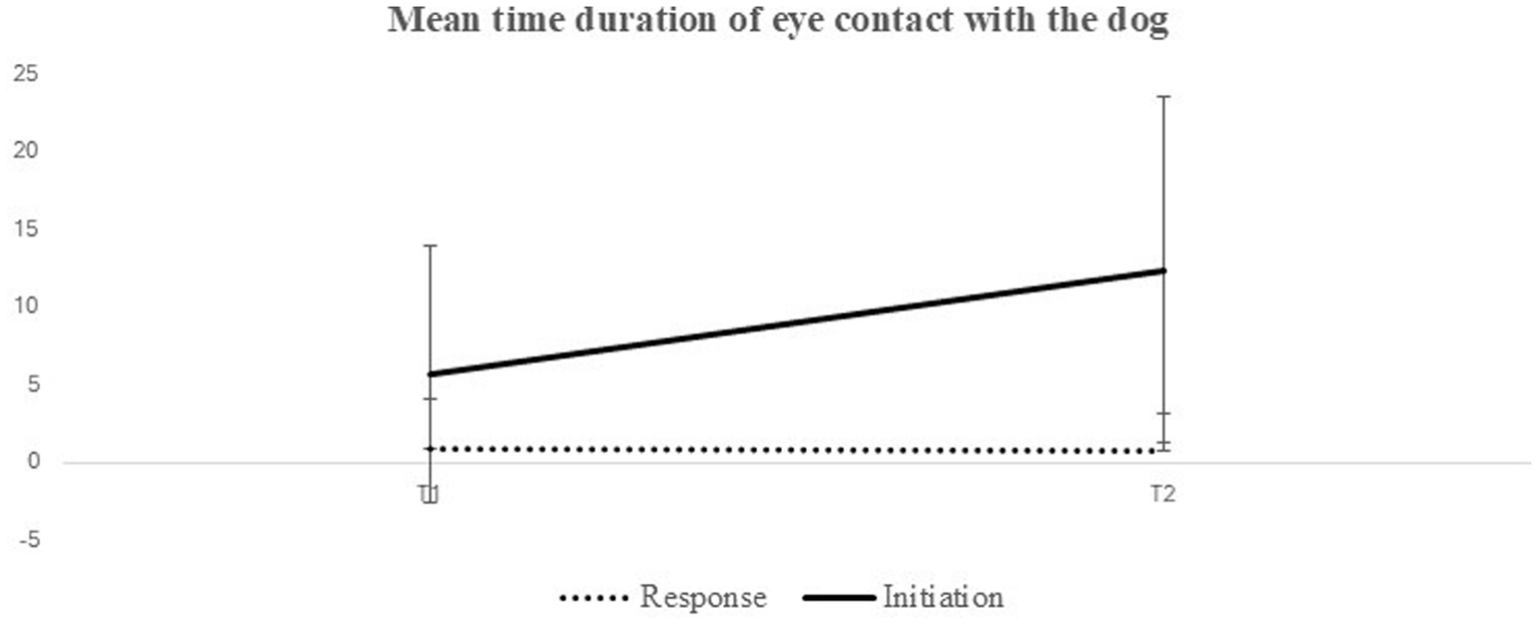
Mean duration of eye contact with the dog at the start and the end of DTI.

We then examined changes in the average duration of eye contact with the dog at the beginning and conclusion of the DTI period. A 2 × 2 ANOVA with repeated measures for Time was conducted, revealing significant effects for both time [*F*(1,36) = 14.44, *p* < 0.001, *η*^2^ = 0.28] and behavior mode [*F*(1,36) = 33.10, *p* < 0.001, *η*^2^ = 0.47]. Additionally, a significant interaction between time and behavior mode was found [*F*(1,36) = 14.14, *p* < 0.001, *η*^2^ = 0.28], indicating that the effect of time on the duration of eye contact varied based on the condition (therapist-directed and participant-initiated) (Figure 2).

Further exploration of this interaction using one-way ANOVAs for each behavior mode separately revealed a significant time effect only for participant-initiated behaviors [*F*(1,36) = 16.59, *p* < 0.001, *η*^2^ = 0.32]. This result indicates that participants’ eye contact duration increased from T1 (*M* = 5.72, SD = 1.36) to T2 (*M* = 12.43, SD = 1.82) when initiating communication with the dog. No significant time effect was noted for the duration of eye contact with the dog in response to the therapist’s instructions [*F*(1,36) = 0.002, *p* = 0.97, *η*^2^ = 0.00], suggesting that the mean eye contact duration remained unchanged when participants responded to the therapists’ instructions. In summary, the findings revealed a significant increase in the average duration of eye contact with the dog by the end of the DTI compared to the start, only for participant-initiated eye contact.

#### 3.2.2 Verbal behaviors

We further assessed participants’ verbal behavior toward the dog in two behavior modes: in response to therapists’ instructions and through participant-initiated interactions. A 2 × 2 (2 times × 2 behavior modes) ANOVA with repeated measures for time was conducted to examine the number of events of giving commands to the dog. The analysis revealed a significant time effect [*F*(1,36) = 38.90, *p* < 0.001, *η*^2^ = 0.52], indicating an increase in the number of commands given by the conclusion of the DTI. However, there was no significant effect for behavior mode [*F*(1,36) = 0.03, *p* = 0.87, *η*^2^ = 0.00], nor a significant interaction between time and behavior mode [*F*(1,36) = 0.80, *p* = 0.38, *η*^2^ = 0.02]. This suggests that the increase in commands occurred irrespective of whether they were in response to therapist instructions or initiated by the child (Table 1).

### 3.3 Behaviors directed toward therapist

Next, changes in non-verbal and verbal communication behaviors directed toward the therapist over time were assessed through two 2 × 2 MANOVAs with repeated measures for time (T1, T2) and behavior mode (responsiveness to therapists’ instructions and participant-initiated) as independent variables.

#### 3.3.1 Non-verbal behaviors

**Figure 3:**
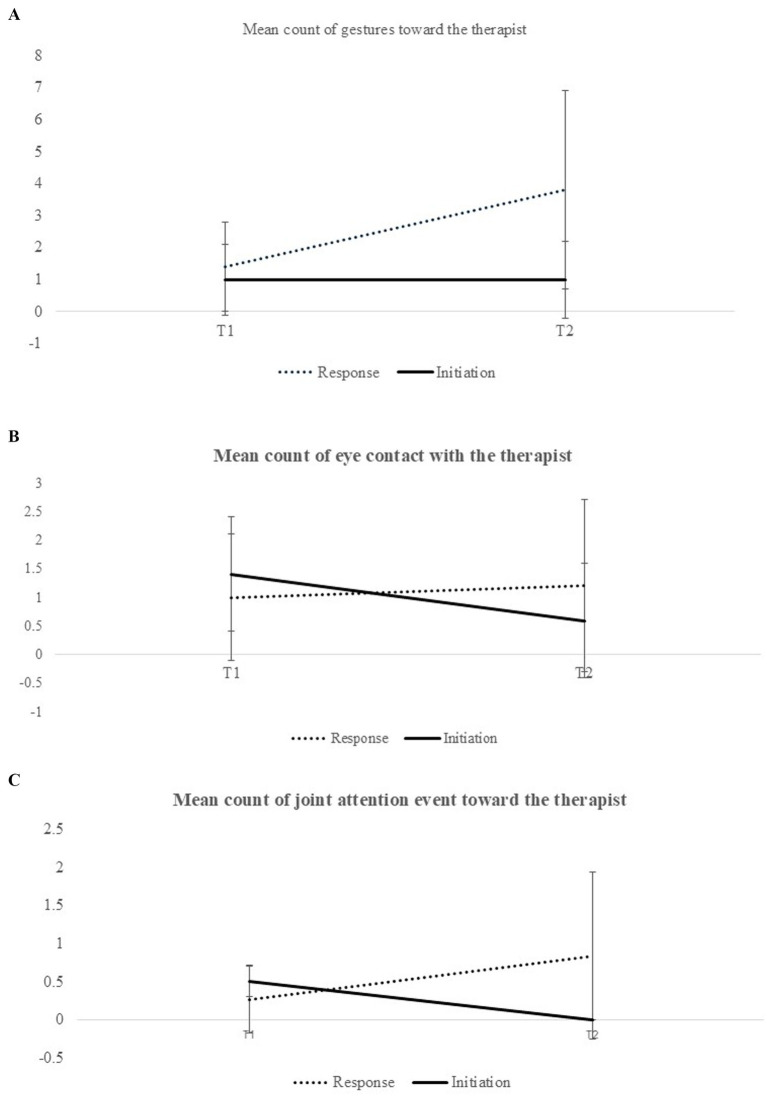
**(A)** Mean number of participant-initiated and responsive eye contact events toward the therapists at the start and the end of DTI. **(B)** Mean number of participant-initiated and responsive gesture events toward the therapists at the start and the end of DTI. **(C)** Mean number of participant-initiated and responsive joint attention events toward the therapists at the start and the end of DTI.

The analysis for non-verbal behaviors yielded a significant time effect [*F*(7,30) = 4.036, *p* = 0.003, *η*^2^ = 0.485], a significant behavior mode effect [*F*(7,30) = 8.403, *p* < 0.001, *η*^2^ = 0.662] and a significant interaction between time and behavior mode [*F*(7,30) = 4.554, *p* = 0.001, *η*^2^ = 0.515]. Two-way ANOVAs for each behavior revealed significant interactions for the counts of eye contact [*F*(1,36) = 6.67, *p* < 0.05, *η*^2^ = 0.15], gestures [*F*(1,36) = 18.75, *p* < 0.001, *η*^2^ = 0.34], and joint attention events [*F*(1,36) = 9.76, *p* < 0.05, *η*^2^ = 0.21] (see Figures 3A–C).

We then conducted one-way ANOVAs separately for both behavior modes, responses to therapists’ instructions, and participant-initiated behaviors. Concerning responses to therapists, significant time effects were observed for gestures [*F*(1,36) = 23.74, *p* < 0.001, *η*^2^ = 0.39] and joint attention events [*F*(1,36) = 9.11, *p* = 0.005, *η*^2^ = 0.20], indicating an increased number of gestures and joint attention events from T1 to T2. However, the change in mean number of eye contact events did not show a significant time effect [*F*(1,36) = 0.4, *p* = 0.55, *η*^2^ = 0.11]. For participant-initiated behaviors, a significant time effect was noted [*F*(1,36) = 6.8, *p* = 0.013, *η*^2^ = 0.16], with fewer eye contact events noted after the DTI. However, no significant time effects were observed for gestures [*F*(1,36) = 0.05, *p* = 0.84] or joint attention [*F*(1,36) = 2.06, *p* = 0.16].

**Figure 4:**
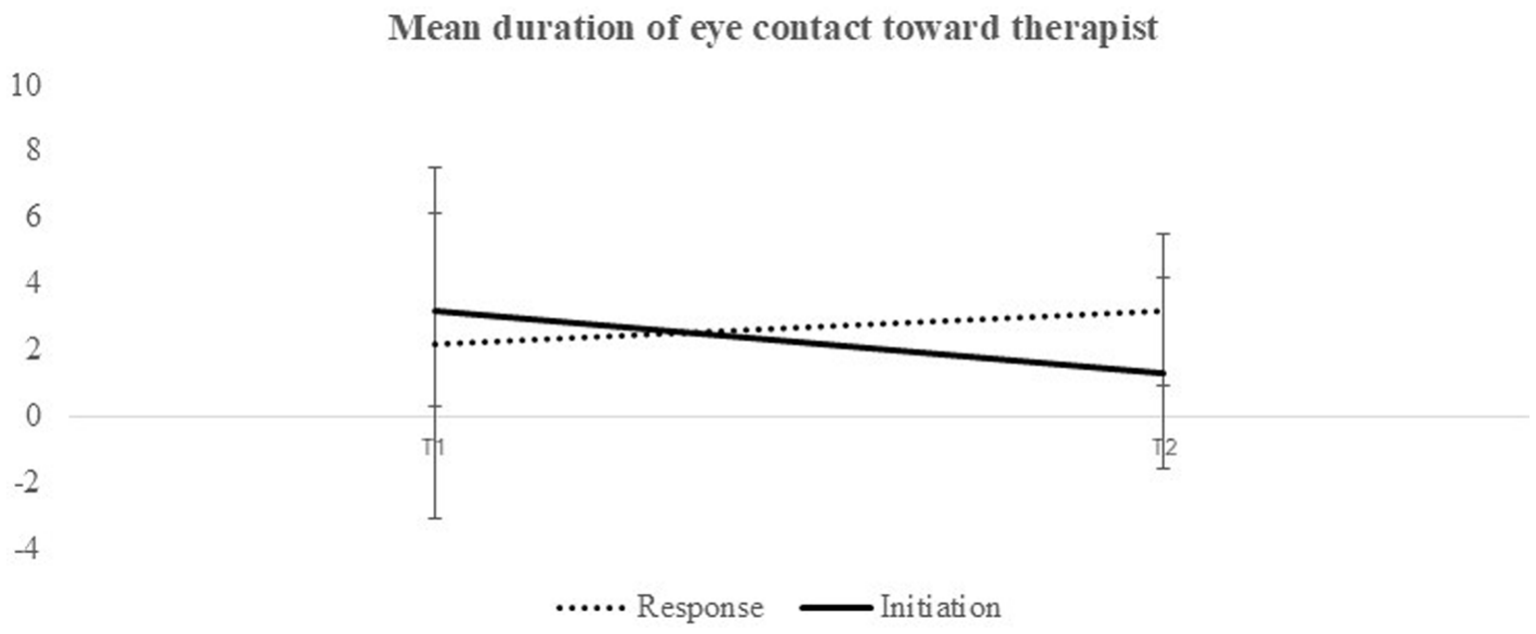
Mean duration of participant-initiated and responsive eye contact with the therapist at the start and the end of DTI.

A two-way ANOVA analyzing the duration of eye contact highlighted a significant interaction between time and behavior mode [*F*(1,36) = 4.79, *p* = 0.035, *η*^2^ = 0.11]. No significant time effect [*F*(1,36) = 3.37, *p* = 0.07, *η*^2^ = 0.08], or behavior mode effect were noted [*F*(1,36) = 0.01, *p* = 0.93]. Subsequent one-way ANOVAs for each behavior mode revealed no significant time effect for the duration of eye contact during responses to the therapists’ instructions [*F*(1,36) = 0.002, *p* = 0.96]. However, when participants initiated eye contact, a significant time effect emerged [*F*(1,36) = 6.73, *p* = 0.014, *η*^2^ = 0.15], indicating a shorter duration of eye contact by the end of the DTI compared to its commencement (see Figure 4).

#### 3.3.2 Verbal communication

A two-way MANOVA with repeated measures for time was conducted to assess changes in verbal communication behaviors, with time (T1, T2) and behavior mode as independent variables. The analysis yielded a significant time effect [*F*(6,31) = 2.52, *p* = 0.05, *η*^2^ = 0. 28].

Subsequent one-way ANOVAs targeting specific verbal behaviors identified a significant time effect for sharing behaviors and asking and responding to questions. While asking and responding to questions increased during the intervention period, sharing behaviors decreased by the conclusion of the DTI program (see Table 2). However, sharing behaviors were also rare at the beginning of the intervention. Additionally, a significant behavior mode effect was found [*F*(6,31) = 8.173, *p* < 0.001, *η*^2^ = 0.61]. However, the time and behavior mode interaction was insignificant [*F*(6,31) = 1.71, *p* = 0.16, *η*^2^ = 0.21].

**Table 2:** Counts of verbal behaviors directed toward the therapists at the start (T1) and end (T2) of DTI.

	T1 *M* (SD)	T2 *M* (SD)	*F*(1,36)	*p*	*η* ^2^
Verbal behaviors					
Statement	0.81 (0.19)	0.63 (0.11)	0.75	0.39	0.02
Question/response	0.86 (0.17)	1.37 (0.27)	3.06	0.09	0.07
Request	0.23 (0.69)	0.17 (0.05)	0.52	0.47	0.03
Refusal	0.24 (0.1)	0.08 (0.03)	2.33	0.13	0.04
Sharing	0.09 (0.03)	0.0	8.4	**0.006**	0.18

Values in bold are statistically significant.

### 3.4 Total number of verbal, non-verbal, and maladaptive behaviors

**Figure 5:**
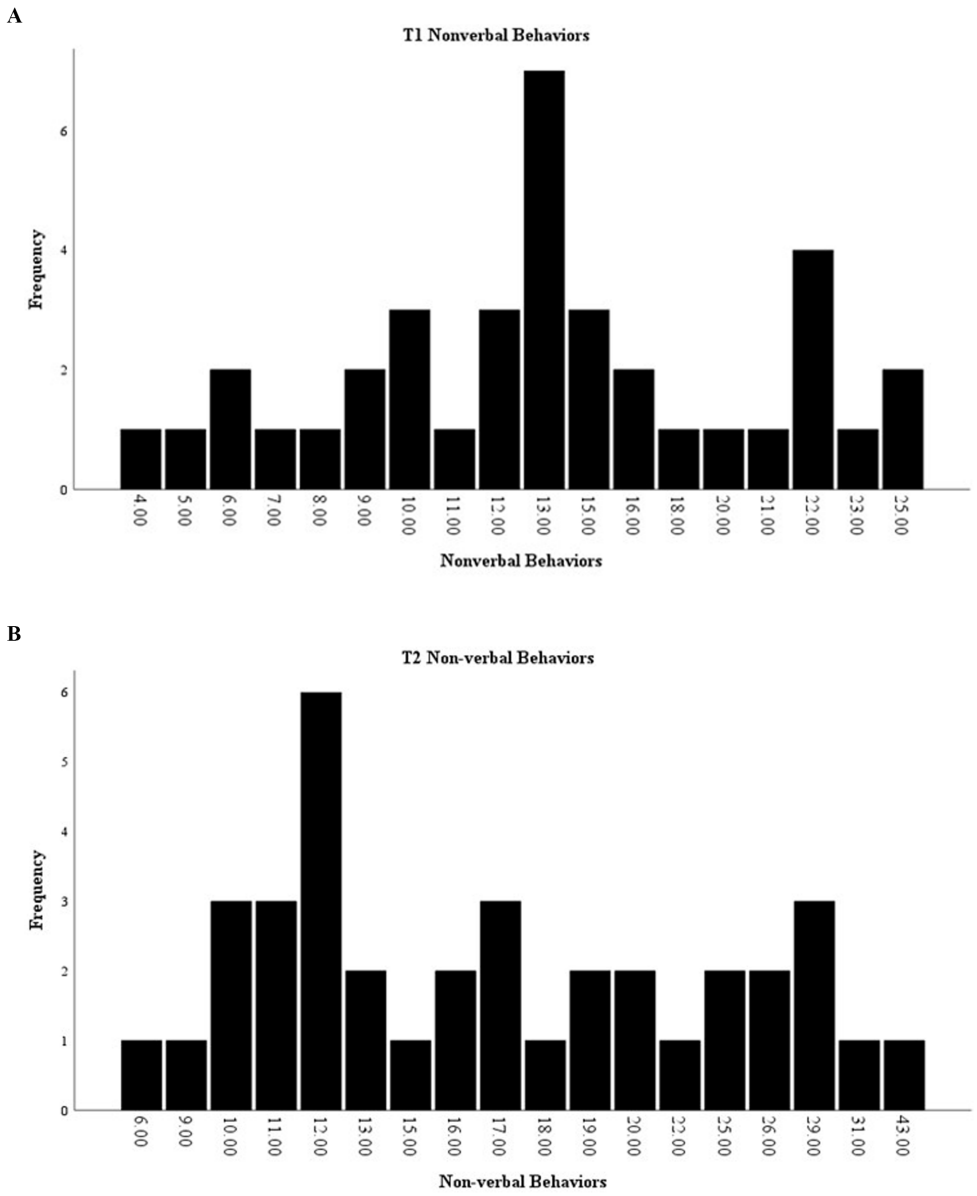
**(A)** Distribution of T1 counts of nonverbal behaviors. **(B)** Distribution of T2 counts of nonverbal behaviors.

In addition to specific behaviors, the total (toward the dog and therapist; initiated and responsive) of verbal, non-verbal, and maladaptive behaviors were compared before and after the intervention. Total non-verbal behaviors showed a significant effect of time [*F*(1,36) = 7.15, *p* = 0.01], with an increase in non-verbal behaviors from the start of the intervention (*M* = 14.03, SD = 5.80) to its completion (*M* = 17.70, SD = 7.92). The range for non-verbal behaviors at T1 was 4–25, with a median of 13 (Figure 5A), and at T2, the range was 6–43, with a median of 16 (Figure 5B).

**Figure 6:**
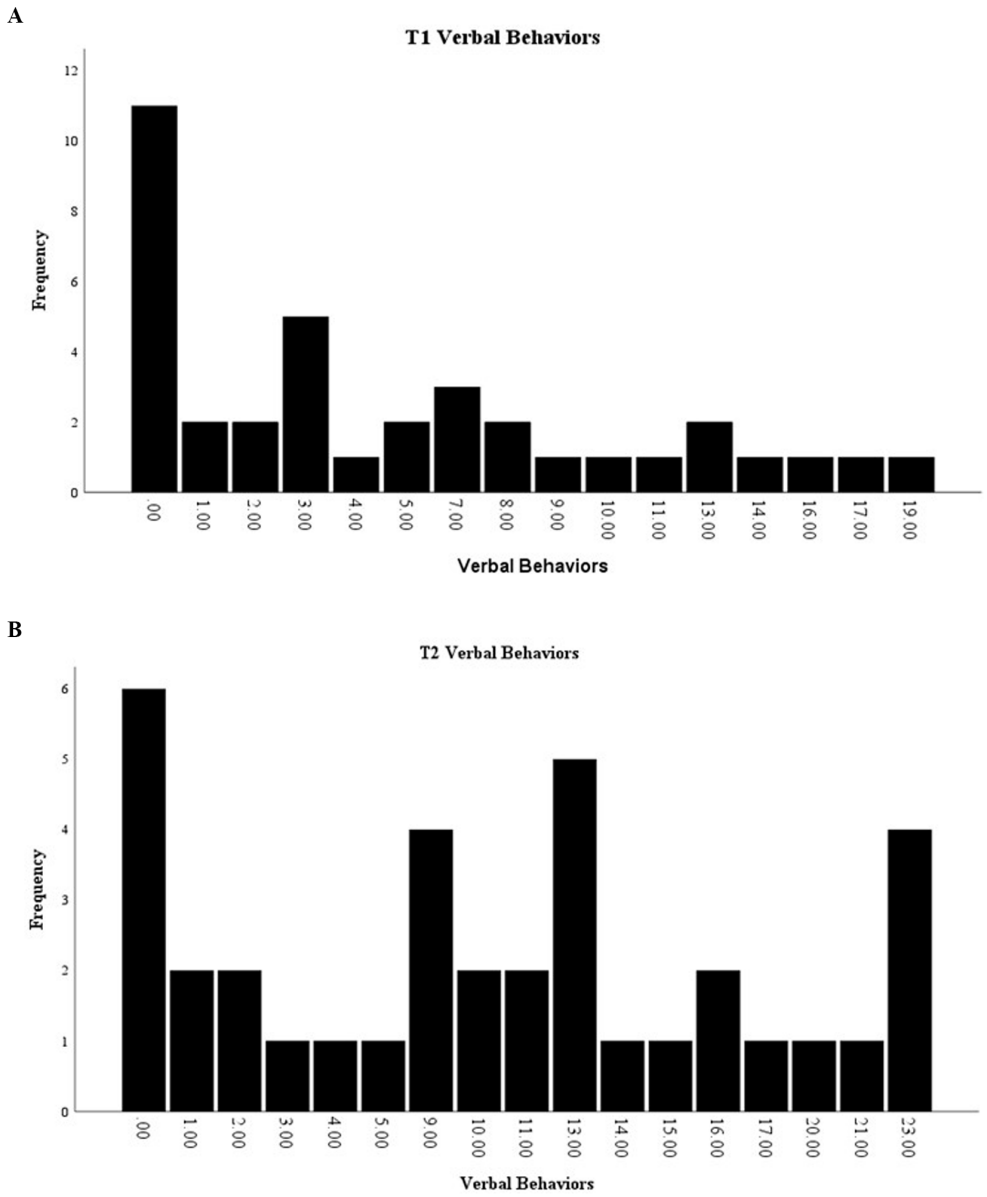
**(A)** Distribution of T1 counts of verbal behaviors. **(B)** Distribution of T2 counts of verbal behaviors.

Total verbal behaviors also demonstrated a significant effect of time [*F*(1,36) = 5.24, *p* < 0.001], with verbal behaviors increasing from the start of the intervention (*M* = 5.24, SD = 5.58) to the end (*M* = 10.05, SD = 7.65). At T1, the range for verbal behaviors was 0–19, with a median of 3 (Figure 6A), while at T2, the range expanded to 0–23, with a median of 10 (Figure 6B).

**Figure 7:**
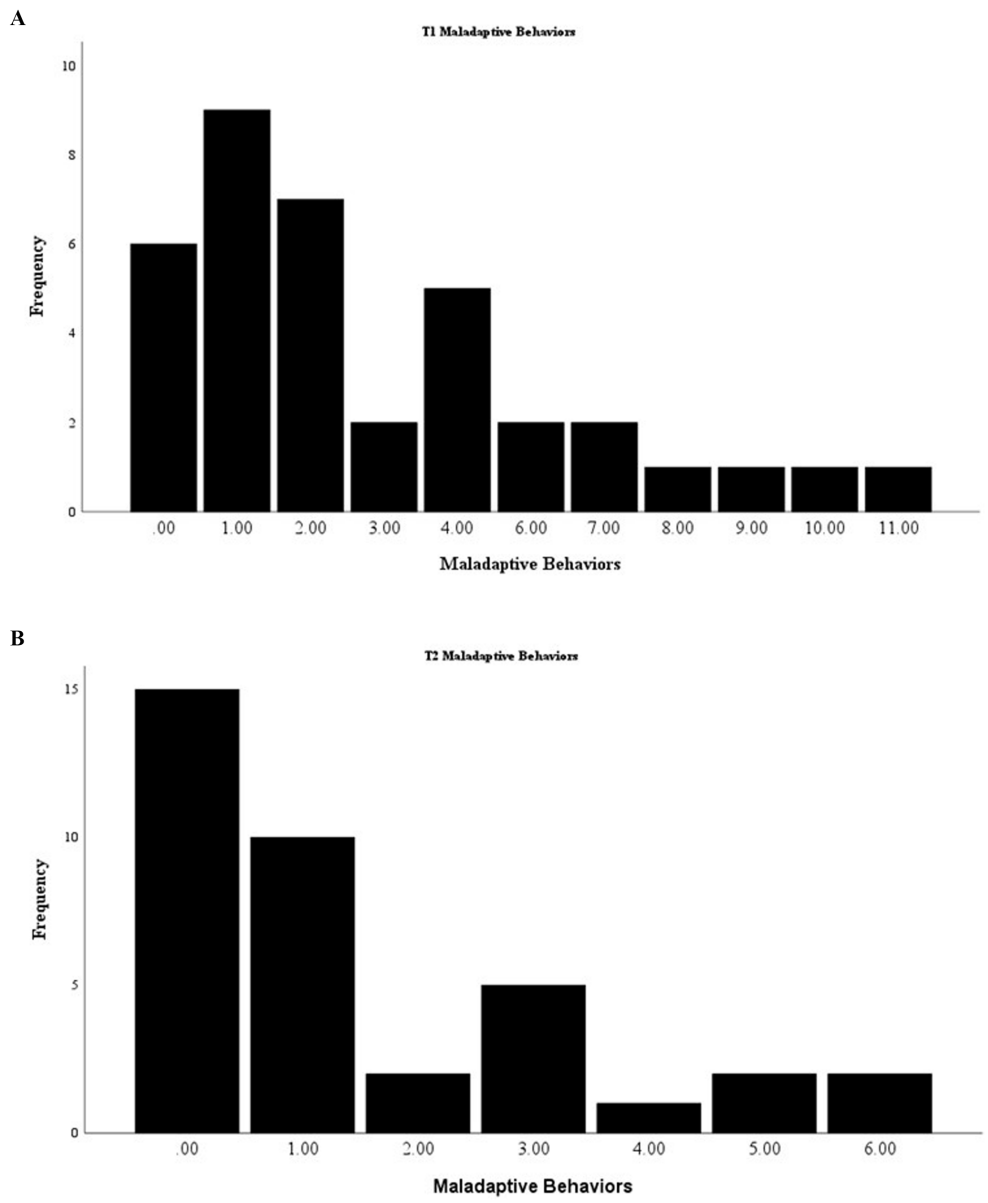
**(A)** Distribution of T1 counts of maladaptive behaviors. **(B)** Distribution of T2 counts maladaptive behaviors.

Total maladaptive behaviors showed a significant time effect as well [*F*(1,36) = 10.45, *p* = 0.003], with a decrease in maladaptive behaviors from the start of the intervention (*M* = 3.05, SD = 3.01) to its completion (*M* = 1.49, SD = 1.82). The range for maladaptive behaviors at T1 was 0–11, with a median of 2 (Figure 7A), and at T2, the range was reduced to 0–6, with a median of 1 (Figure 7B).

### 3.5 Maladaptive behaviors

We further investigated maladaptive behaviors directed toward either the therapists or the dogs. A one-way MANOVA with repeated measures for time revealed a significant time effect [*F*(5,32) = 3.001, *p* = 0.025, *η*^2^ = 0.319]. Subsequent one-way ANOVAs for individual maladaptive behaviors revealed significant time effects for inappropriate physical contact with the dog or the therapist and repetitive motor movements (Table 3). These inappropriate behaviors declined by the end of the DTI compared to its onset.

**Table 3:** Counts of maladaptive behaviors at the start (T1) and end (T2) of DTI.

	T1 *M* (SD)	T2 *M* (SD)	*F*(1,36)	*p*	*η* ^2^
Maladaptive behaviors					
Inappropriate physical contact	0.75 (0.27)	0.08 (0.04)	**6.46**	**0.01**	0.15
Inappropriate facial expressions	0.0	0.16 (0.09)	3.17	0.08	0.08
Repetitive motor movements	1.59 (0.33)	0.89 (0.26)	**5.88**	0.02	0.14
Maladaptive sensory behaviors	0.51 (0.17)	0.32 (0.11)	2.2	0.14	0.07
Repetitiveness with objects	0.16 (0.09)	0.02 (0.02)	1.97	0.16	0.05

Values in bold are statistically significant.

Maladaptive verbal behaviors, repetitive behaviors, unusual intonations, and stereotyped language were also noted. However, their occurrence was sporadic, and statistical analysis could not be conducted.

### 3.6 Relationship between participants’ characteristics and DTI progress

We subsequently explored the relationship between participants’ characteristics and their progress in targeted behaviors. To quantify progress, we summed the scores for communicative and social behaviors at Time 1 (T1) and Time 2 (T2). These behaviors were categorized into two groups: responses to the therapists’ instructions and behaviors initiated by participants. Progress was calculated by subtracting T1 scores from T2 scores for compliant and self-initiated behavior measures. The participants’ characteristics considered included age, cognitive abilities (as measured by WPPSI scores), severity of autism (SRS-II scores), and anxiety levels (SCAS scores) (Table 4).

**Table 4:** Correlation between the participant’s characteristics and progress in targeted behavior.

	T2–T1 social-communication behaviors occurring in response to therapists’ instructions		T2–T1 self-initiated social-communication behaviors	
	*r*	*p*	*r*	*p*
Age	−0.15	0.18	−0.08	0.30
WPPSI-III scores	**0.48**	**0.001**	**0.46**	**0.002**
SRS-II scores	**−0.45**	**0.003**	**−0.23**	**0.09**
SCAS scores	−0.13	0.21	0.1	0.27

Values in bold are statistically significant.

Pearson correlation analyses revealed a moderate negative correlation between SRS scores and improvements in response to the therapists’ instructions. This indicates that participants with less severe autism symptoms responded more to therapists’ directions. Additionally, a significant moderate positive correlation was observed between WPPSI scores and improvements in responsiveness to the therapist, suggesting that children with higher cognitive abilities demonstrated more pronounced enhancements. Interestingly, neither age nor SCAS scores significantly correlated with improved response to the therapists’ instructions.

## 4 Discussion

Our previous research demonstrated significant improvements in adaptive social and communication skills among children with autism who participated in the Dog Training Intervention (DTI) using a controlled cross-over study design (Ben-Itzchak and Zachor, 2021).

### 4.1 The current study’s findings and explanations

The present study extended these findings by exploring subtle changes in social communication behaviors following DTI. Specifically, we examined verbal and non-verbal behaviors observed in video recordings from the initial and final intervention sessions, focusing on two dimensions: responses to the therapist’s instructions and self-initiated behaviors, and across two conditions: interactions with the dog and interactions with the therapist.

The current findings revealed increased non-verbal communication post-DTI, including more frequent and prolonged eye contact, gestures, and facial expressions, and increased verbal commands directed toward the dog. Notably, the rise in eye contact with the dog was primarily self-initiated (not prompted by the therapist), underscoring the potential of DTI to enhance spontaneous communication. This supports the idea that the DTI, which emphasizes handling the dog, fosters verbal and non-verbal communication and enhances self-initiated communication, especially toward the dog. Moreover, improvements were observed in the number of gestures, joint attention, and question/answer behaviors toward the therapist following the intervention. However, the rise in gestures and joint attention behaviors was more likely to occur in response to therapist prompts rather than being self-initiated by the children. While the DTI fostered verbal and non-verbal communication toward adults, these interactions were largely prompt-dependent.

A reduction in self-initiated eye contact, shorter duration of eye contact, and verbal sharing with the therapist was also noted. The findings indicate that interactions with the dog led to improvements and increased social behaviors, including more frequent and prolonged self-initiated eye contact. In contrast, interactions with the adult showed improvements in behaviors (gestures and joint attention) only in response to instructions, while self-initiated behaviors such as eye contact decreased in frequency and duration and in sharing. It appears that during the intervention, the child’s focus shifted from the adult to the dog, with the child more successfully initiating prosocial behaviors with the dog. This may be the desired process in this type of intervention; however, it would be beneficial in the future to incorporate tasks that also require the child to initiate interactions with the adult. Practicing both verbal and non-verbal communication with the therapist should be integrated into the program. Additionally, working with a pair or group of children may enhance social behaviors toward humans, which ultimately remains the primary goal. Tasks that encourage requests and questions would help achieve this aim.

The current study’s findings build on existing knowledge of social communication challenges in ASD, which frequently involve reduced use of joint attention and self-initiated gestures. Previous research highlights that children with ASD tend to use gestures primarily for requesting rather than sharing attention, which limits their opportunities for social engagement and impacts language development over time (Valle et al., 2021). In this study, DTI addressed these specific areas by emphasizing spontaneous social behaviors, particularly through interactions with the dog. One of the significant findings of this study was a reduction in maladaptive behaviors, including inappropriate physical contact with both the dog and the therapist, as well as fewer repetitive motor movements. Importantly, no direct intervention was applied to address maladaptive behaviors, yet the structured encounters with the dog had a positive impact. This highlights the broader therapeutic potential of DTI beyond its primary focus on social communication skills.

### 4.2 Correlations of individual characteristics and response to DTI

The correlation between children’s characteristics and their progress in social communication behaviors following DTI revealed that children with less pronounced autism and higher cognitive abilities were associated with greater responsiveness to the therapist’s directions. This suggests that those with better cognitive abilities may have a greater capacity to understand instructions and acquire new skills. Additionally, children with fewer impairments in social skills and less pronounced repetitive and restricted behaviors exhibited more self-initiated social communication, possibly were less preoccupied with stereotypic behaviors, and could better generalize learned skills in different situations. These findings underscore the importance of considering individual differences when designing and implementing DTI, as they can help identify who stands to benefit the most from the intervention and inform necessary adaptations for children with more severe impairments.

### 4.3 Comparison to previous research

While there is limited research on the effectiveness of dog intervention programs in autism, most studies, including ours, have reported improvements in social-communication behaviors. The current study is among the few that specifically examined detailed changes in non-verbal and verbal behaviors directed toward the dog or the therapist. Additionally, this study is unique in distinguishing between self-initiated behaviors and those prompted by the therapist’s instructions.

The findings of the current study both align with and diverge from those of several previous investigations, highlighting similarities and nuances in the effects of dog-assisted interventions on children with autism. Consistent with our findings, prior studies have reported increases in prosocial behavior (Redefer and Goodman, 1989) and improvements in non-verbal (Ávila-Álvarez et al., 2020; Germone et al., 2019; Martin and Farnum, 2002) and verbal interactions (Ávila-Álvarez et al., 2020; Fung and Leung, 2014; Martin and Farnum, 2002). Of them, several researchers observed increased social-communication behaviors directed toward both the dog and the adult (Germone et al., 2019; Ávila-Álvarez et al., 2020), while others noted improvements only in interactions with the dog (Redefer and Goodman, 1989). Notably, Martin and Farnum (2002) found reduced responsiveness to therapist questions in the presence of a live dog. Additionally, reductions in stereotypical behaviors were reported in multiple studies (Fung and Leung, 2014; Redefer and Goodman, 1989). Align with Barcelos et al. (2021), the improvement in these various aspects may be attributed to dogs providing a consistent source of non-judgmental companionship and enhancing social engagement.

These findings suggest that different aspects of social communication may be variably impacted depending on the specific design and context of the intervention. A unique feature of the current study is introducing a dog-training approach, where children are taught to act as dog trainers, which results in positive outcomes. However, one of the key gaps across most studies is the limited exploration of individual differences in children’s responses to dog-assisted interventions. While Germone et al. (2019) found no association between non-verbal IQ and behavioral outcomes, our study identified that higher cognitive ability and lower levels of ASD-related impairments were associated with better outcomes. This suggests that individual characteristics may play a crucial role in moderating the effectiveness of these interventions. Although many benefits to the human-dog bond for individuals with autism were previously reported, some challenges and negative impacts have also been noted. For certain autistic individuals, sensory sensitivities can cause discomfort or anxiety in response to barking or physical contact with dogs (Barcelos et al., 2021).

### 4.4 The strengths and weaknesses of the study

The current study offers several strengths. It adds to the limited research examining behavioral changes during dog-assisted interventions for autistic children and adults. The study addresses several weaknesses previously noted in AAI research (O’Haire 2013, 2017; Rehn et al., 2023), including a larger sample size than most studies of this type, a smaller participants’ age range, and a clear protocol. What sets this study apart is its focus on both verbal and non-verbal communicative behaviors, as well as maladaptive behaviors, while considering both the recipient of the behaviors (dog or therapist) and whether the communication was self-initiated or in response to therapist prompts. Additionally, the study introduces a unique dog-training protocol designed to teach children how to handle a dog. The observed improvements across various measures suggest that the existing protocol is effective and could be expanded to higher levels of dog training. Moreover, introducing new elements—such as enhancing facial expressions and verbal sharing—may address fewer areas for improvement. Integrating pair-based activities where children interact by giving instructions or reporting to one another could further enhance the intervention.

Another strength of the study is its integration within an educational framework, demonstrating the feasibility of implementing the program in existing educational settings. Additionally, the relatively narrow age range of participants, which included only preschool children, enables the conclusion of intervention goals for children of this young age. Previous studies (except Ávila-Álvarez et al., 2020) included broader age ranges, allowing for more generalizable conclusions across different age groups.

Several limitations should be noted. While this type of study is typical, the absence of a control group presents a limitation. The original study included a control group that did not receive the intervention. However, for this video analysis, including a control group was neither feasible, logical, nor ethical, as filming the children with the dog without prior interaction was impractical.

Another limitation was the exclusion of many videos due to strict conditions regarding camera angles and video quality, which left only 37 children (out of the original 73) in the final analysis. While this sample size is relatively large compared to similar studies, greater care during filming could have yielded a larger dataset. An additional limitation is being one of the coders unblind to the research condition. However, the high correlation with a second blind coder (91%) and the negative results in some respects may ensure unbiased results.

The present study contributes to the growing body of evidence supporting the effectiveness of dog-assisted interventions for children with autism. Importantly, it highlights the nuanced changes in social-communication behaviors, particularly the distinction between self-initiated behaviors and those prompted by therapists. Overall, the positive findings of this study, consistent with previous research on the effectiveness of dog training interventions for young autistic children, support the recommendation to integrate similar programs alongside conventional intervention approaches. Future research should explore ways to enhance social initiation toward therapists and peers, in addition to the dog, and consider tailoring interventions to the child’s characteristics, such as cognitive ability and the level of autism characteristics.

## Data Availability

The datasets presented in this article are not readily available because the data originates from video recordings of the children, and due to confidentiality, it is not possible to share the videos, or the data derived from them. Requests to access the datasets should be directed to benitze@ariel.ac.il.
